# Density-Dependent Effects of Invasive *Pomacea canaliculata* on Nutrient Status, Enzyme Activities, and Bacterial Community Structure in Flooded Paddy Soil Microcosms

**DOI:** 10.3390/microorganisms14091895

**Published:** 2026-08-26

**Authors:** Liang Guo, Yinghan Liu, Yijun Weng, Liangliang Hu, Tan Ke, Yuqin Mao, Yin Lu

**Affiliations:** 1 Key Laboratory of Pollution Exposure and Health Intervention of Zhejiang Province, College of Biological and Environmental Engineering, Zhejiang Shuren University, Hangzhou 310015, China; liangguo93@126.com (L.G.);; 2 College of Life Sciences, Zhejiang University, Hangzhou 310058, China; 3 Chun’an County Meteorological Bureau, Hangzhou 311700, China

**Keywords:** golden apple snail, biological invasion, paddy soil microbiome, nutrient cycling, soil enzyme activity

## Abstract

The invasive golden apple snail (*Pomacea canaliculata*) threatens rice agroecosystems, yet its direct density-dependent effects on flooded paddy soil biogeochemistry and bacterial communities remain unclear. We established flooded soil microcosms with four snail densities (0, 2, 4, and 6 snails/box) for 20 days, without external food inputs. Soil dissolved organic carbon (DOC), ammonium nitrogen (NH_4_^+^-N), nitrate nitrogen (NO_3_^−^-N), and the activities of β-glucosidase, N-acetyl-β-D-glucosaminidase, urease, and dehydrogenase were measured, and bacterial communities were characterized by full-length 16S rRNA gene amplicon sequencing. Snail density was significantly and positively related to all three nutrient variables and all four enzyme activities. The dominant bacterial phyla and genera remained stable, and bacterial α-diversity changed little among treatments, despite a small but significant increase in Simpson diversity in the high-density treatment. PERMANOVA detected significant differences in overall bacterial community structure among density treatments, while environmental fitting identified DOC, urease, and dehydrogenase as variables significantly associated with community variation. These findings indicate that living golden apple snails can alter nutrient availability, soil biochemical activity, and bacterial community organization in flooded paddy soil, revealing a belowground pathway through which this invader may influence paddy ecosystem functioning.

## 1. Introduction

The invasive golden apple snail (*Pomacea canaliculata*) is one of the most destructive freshwater mollusks in rice-growing regions worldwide [1]. Owing to its high reproductive capacity, broad dietary niche, rapid dispersal, and remarkable environmental adaptability, this species has successfully colonized tropical and subtropical regions across Asia and continues to expand its potential distribution under ongoing climate change [2,3]. As a major rice pest and invasive alien species, golden apple snail has caused substantial ecological and economic losses by damaging rice seedlings, reducing aquatic vegetation, altering wetland biodiversity, and threatening agricultural production [1].

However, invasive freshwater animals are not merely consumers within food webs. Increasing evidence suggests that they may also function as ecosystem engineers capable of modifying habitat conditions and regulating ecosystem processes [4]. Through feeding, excretion, egestion, and bioturbation, aquatic mollusks can influence nutrient retention, organic matter decomposition, sediment characteristics, and material cycling at the water–soil interface [5,6]. Therefore, evaluating the ecological consequences of golden apple snail invasion requires moving beyond direct herbivory and considering its potential effects on nutrient dynamics and belowground microbial processes in paddy ecosystems.

Golden apple snail spends most of their life on the sediment surface or within shallow flooded soils and can therefore substantially modify the physicochemical environment of paddy fields [1]. Mesocosm experiments conducted in wetlands and shallow freshwater ecosystems have consistently shown that golden apple snail reduces aquatic macrophyte biomass, increases dissolved carbon and nitrogen concentrations, and shifts primary production from benthic to pelagic pathways [5,6,7,8]. These effects can vary with nutrient availability, habitat characteristics, and co-occurring invasive species, highlighting their context dependence [6,7,9].

Compared with the extensive knowledge regarding aquatic vegetation and water quality, much less is known about how golden apple snail influences soil environments. Because flooded paddy soils represent hotspots of microbial activity and nutrient transformation, even subtle changes in carbon and nitrogen availability may substantially influence microbial metabolism, extracellular enzyme activities, and microbial community assembly. Understanding whether golden apple snail modifies these belowground processes is therefore essential for evaluating their ecosystem-level impacts.

Long-term field observations have shown that freshwater snail activity can alter soil organic matter and the availability of nitrogen, phosphorus, and potassium [10]. Furthermore, incorporation of golden apple snail biomass as an organic amendment significantly increases soil organic carbon and nutrient availability, stimulates extracellular enzymes such as N-acetyl-β-D-glucosaminidase (NAG), and reshapes bacterial community composition [11,12,13]. Investigations using live golden apple snails have also reported changes in dissolved organic carbon (DOC) and available nitrogen in saline agricultural soils [14]. More recently, microcosm studies have demonstrated that golden apple snail can modify the composition, functional potential, and assembly processes of environmental biofilm-associated bacterial communities while facilitating the dispersal of gut microorganisms into surrounding habitats [15].

Nevertheless, most previous studies have investigated decomposing snail biomass, saline soils, natural wetlands, or aquatic biofilms rather than typical flooded paddy soils. In particular, it remains unclear whether living golden apple snails can directly regulate soil biogeochemical processes in the absence of rice plants and external food inputs, and whether these effects exhibit density dependence. Because invasion intensity often determines ecological impacts, clarifying density-dependent responses is essential for predicting ecosystem consequences under different invasion scenarios.

To address these questions, we established flooded paddy soil microcosms containing different densities of living golden apple snails while excluding rice plants and external food sources. Soil ammonium (NH_4_^+^-N), nitrate (NO_3_^−^-N), dissolved organic carbon (DOC), and the activities of β-glucosidase (BG), urease, dehydrogenase, and N-acetyl-β-D-glucosaminidase (NAG) were quantified, and bacterial diversity and community composition were characterized using high-throughput sequencing. We further identified the environmental variables most strongly associated with bacterial community shifts. We hypothesized that these soil biochemical and bacterial community responses would vary with snail density. By disentangling the direct effects of living apple snails on flooded paddy soils, this study provides a controlled assessment of their short-term belowground ecological effects and improves our understanding of biological invasions in rice agroecosystems.

## 2. Materials and Methods

### 2.1. Soil and Snail Collection

Soil was collected from the 0–15 cm plough layer of a typical paddy field in Zhejiang Province, China. Sampling locations near field ridges, drainage ditches, and areas with anomalous water accumulation were avoided. Soil was collected from multiple points within the same field, thoroughly homogenized, and transported to the laboratory. Stones, plant residues, and visible soil macrofauna were manually removed, after which the soil was homogenized and maintained in a fresh condition until use. To preserve the original microbial community as much as possible, the soil was neither air-dried nor sterilized. Initial soil properties, including soil organic carbon (SOC), total nitrogen (TN), ammonium nitrogen (NH_4_^+^-N), and nitrate nitrogen (NO_3_^−^-N), were determined before the experiment.

Golden apple snails (*Pomacea canaliculata*) were collected from paddy fields or associated irrigation and drainage channels in Zhejiang Province. Subadult or adult individuals from the same collection batch were selected based on similar body size, normal activity, and intact shells. Shell height and fresh weight were recorded for each individual before the experiment, and individuals of comparable size were used to minimize variation associated with snail size. The collected snails were acclimated indoors for 7 days in dechlorinated tap water under shallow-water conditions. Before introduction into the experimental microcosms, the snails were fasted for 24 h to minimize the influence of gut contents during the initial stage of the experiment.

### 2.2. Experimental Design

A controlled indoor microcosm experiment was conducted with four snail-density treatments: (1) a snail-free control (CK, 0 individuals per microcosm), (2) low density (L, 2 individuals per microcosm), (3) medium density (M, 4 individuals per microcosm), and (4) high density (H, 6 individuals per microcosm). Each treatment consisted of three biological replicates, resulting in a total of 12 microcosms.

Rectangular plastic containers measuring 29.3 cm × 19.8 cm × 14.5 cm were used as experimental microcosms. Each container received fresh soil equivalent to 3.5 kg of dry soil, forming a soil layer approximately 5 cm deep. Dechlorinated tap water was then slowly added along the inner wall of each container to establish a stable overlying water layer of approximately 3 cm, thereby simulating flooded paddy soil conditions.

The microcosms were pre-equilibrated for 7 days without snails or additions of external organic matter or nutrients to allow the soil environment to stabilize. After pre-equilibration, snails were introduced at the designated densities on Day 8, which was defined as experimental Day 0.

No rice plants, external feed, or plant residues were added throughout the experiment. This design excluded plant rhizosphere effects and external carbon and nitrogen inputs, allowing the direct effects of snail activity, excretion, egestion, and bioturbation on the paddy soil microenvironment and microbial processes to be evaluated.

### 2.3. Incubation Conditions and Microcosm Maintenance

The experiment was conducted in a screenhouse at Zhejiang University, China. Each microcosm was covered with fine mesh to prevent snail escape while allowing air exchange. The overlying water depth was maintained at approximately 3 cm throughout the experiment, and evaporative losses were compensated for by adding dechlorinated water to the original water level.

Snail survival and activity were checked every 2–3 days. Individuals that died within 48 h of introduction were immediately replaced with snails of a similar size. Individuals that died more than 48 h after introduction were not replaced, and their bodies were promptly removed to prevent decomposition from affecting water and soil properties. Total snail fresh biomass and the number of surviving individuals in each microcosm were recorded at the beginning (Day 0) and end (Day 20) of the experiment.

### 2.4. Soil Sampling

Soil samples were collected at the end of the 20-day incubation period. One composite soil sample was collected from each microcosm, yielding a total of 12 samples for subsequent chemical, enzymatic, and bacterial community analyses.

Approximately 25–30 g of soil was collected from the upper 0–5 cm layer of each microcosm using a five-point sampling method. The subsamples were combined in sterile sealable bags and thoroughly homogenized. Each composite sample was divided into two aliquots. One aliquot was immediately frozen in liquid nitrogen and subsequently stored at −80 °C for microbial DNA extraction and bacterial community sequencing. The other aliquot was stored at 4 °C for the determination of NH_4_^+^-N, NO_3_^−^-N, dissolved organic carbon (DOC), and soil enzyme activities. Sample pretreatment and the relevant chemical and enzymatic measurements were completed within 48 h of collection.

### 2.5. Soil Chemical Properties and Enzyme Activities

Soil chemical properties and enzyme activities were determined by Nanjing Cavens Testing Technology Co., Ltd. (Nanjing, China). Dissolved organic carbon was extracted with deionized water and quantified using a total organic carbon analyzer. Soil NH_4_^+^-N was extracted with potassium chloride solution and determined using the indophenol blue colorimetric method. Soil NO_3_^−^-N was extracted with potassium chloride solution and measured using a dual-wavelength spectrophotometric method.

The activities of urease (UE), dehydrogenase (DHA), β-glucosidase (BG), and N-acetyl-β-D-glucosaminidase (NAG) were determined using commercial assay kits according to the manufacturers’ instructions. These enzymes were selected to evaluate changes in soil carbon- and nitrogen-related biochemical processes in response to snail activity.

### 2.6. Full-Length 16S rRNA Gene Amplicon Sequencing

Full-length 16S rRNA gene amplicon sequencing was performed by Shanghai GeneCowin Biotechnology Co., Ltd. (Shanghai, China). Total genomic DNA was extracted from soil samples using the FastDNA^®^ SPIN Kit for Soil (MP Biomedicals, Santa Ana, CA, USA) according to the manufacturer’s instructions. DNA integrity was assessed by agarose gel electrophoresis, and DNA concentration was quantified using a Qubit 3.0 fluorometer (Thermo Fisher Scientific, Waltham, MA, USA).

The full-length bacterial 16S rRNA gene was amplified using the primers 27F (5′-AGRGTTYGATYMTGGCTCAG-3′) and 1492R (5′-RGYTACCTTGTTACGACTT-3′), together with 10-bp sample-specific barcodes. Amplicons from different samples, each carrying a unique barcode, were pooled according to the sequencing requirements.

The pooled amplicon library was prepared for Oxford Nanopore Technologies sequencing using the CWseq^®^ DNA Ligation Aid-based Library Prep Kit for Nanopore Sequencing (Cwbio, Taizhou, China) and the Native Barcoding Kit 96 V14 (Oxford Nanopore Technologies, Oxford, UK). The prepared libraries were loaded onto R10.4.1 flow cells and sequenced using the PromethION 2 Solo platform (Oxford Nanopore Technologies, Oxford, UK).

### 2.7. Processing and Taxonomic Classification of ONT Sequencing Data

Raw FASTQ reads were quality- and length-filtered using Chopper v0.12.0 to remove low-quality reads and sequences outside the expected length range [16]. The filtering parameters were set to --quality 10, --minlength 1000, and --maxlength 1800. Subsequent sequence processing was primarily conducted using QIIME 2 version 2023.2 [17].

Primer sequences were removed using the removePrimers function in the DADA2 R package implemented within the QIIME 2 workflow. The VSEARCH plugin in QIIME 2 was subsequently used for sequence dereplication, operational taxonomic unit (OTU) clustering at 97% sequence similarity, and chimera removal. A high-quality OTU abundance table and corresponding representative sequences were generated for downstream analyses.

Taxonomic classification of representative OTU sequences was conducted using the assignTaxonomy function in the DADA2 R package against the SILVA reference database version 138.2 [18]. A confidence threshold of 0.5 was applied during taxonomic assignment.

After quality filtering, primer removal, and chimera removal, 49,841–61,762 non-chimeric reads were retained per sample. To standardize sequencing depth across samples, the OTU table was rarefied to 49,697 reads per sample before diversity and community analyses. The sample-specific sequencing depth and Good’s coverage values are provided in Supplementary Table S1.

The raw full-length 16S rRNA gene amplicon sequencing data generated in this study have been deposited in the NCBI Sequence Read Archive (SRA) under BioProject accession number PRJNA1502501.

### 2.8. Statistical Analyses

All statistical analyses and data visualization were performed in R version 4.5.3. Simple linear regression was used to evaluate relationships between snail density and soil chemical properties, including DOC, NH_4_^+^-N, and NO_3_^−^-N, as well as enzyme activities, including UE, DHA, BG, and NAG.

One-way analysis of variance (ANOVA) was used to test the overall effect of snail-density treatment. When the omnibus ANOVA was significant at *p* < 0.05, Fisher’s protected least significant difference (LSD) test was used for post hoc pairwise comparisons. Because the study employed a balanced design with a limited number of biological replicates, the LSD comparisons were conducted without multiplicity adjustment to avoid an excessive loss of statistical power. Exact *p* values are reported for relevant pairwise comparisons. Fisher’s least significant difference (LSD) test was conducted for post hoc multiple comparisons using the agricolae package.

Pairwise relationships among soil chemical properties and enzyme activities were assessed using Spearman’s rank correlation analysis. Total snail fresh biomass at Day 0 and Day 20 was compared within each density treatment using paired-sample *t*-tests.

Bacterial α-diversity was assessed using the Chao1 richness, Shannon diversity, and Simpson diversity indices calculated from the OTU abundance table. Simple linear regression was used to examine relationships between snail density and each α-diversity index. Differences in α-diversity indices among treatments were evaluated using one-way ANOVA followed by LSD post hoc tests implemented in the agricolae package.

Differences in overall bacterial community structure among treatments were evaluated using permutational multivariate analysis of variance (PERMANOVA) with the adonis2 function and 999 permutations. Principal coordinates analysis (PCoA) based on Bray–Curtis dissimilarities was used to visualize variation in bacterial community structure. Soil variables, including DOC, NH_4_^+^-N, NO_3_^−^-N, UE, DHA, BG, and NAG, were fitted onto the ordination space using the envfit function to assess their associations with bacterial community composition. The homogeneity of multivariate dispersions among treatments was assessed using the betadisper function in the vegan package, based on distances to group spatial medians calculated from the same Bray–Curtis dissimilarity matrix used for PERMANOVA. Significance was evaluated using the permutest function with 9999 permutations. Community analyses were conducted using the vegan package. Statistical significance was accepted at *p* < 0.05.

## 3. Results

### 3.1. Effects of Golden Apple Snail on Soil DOC, NH_4_^+^-N, and NO_3_^−^-N in Flooded Paddy Soil

**Figure 1:**
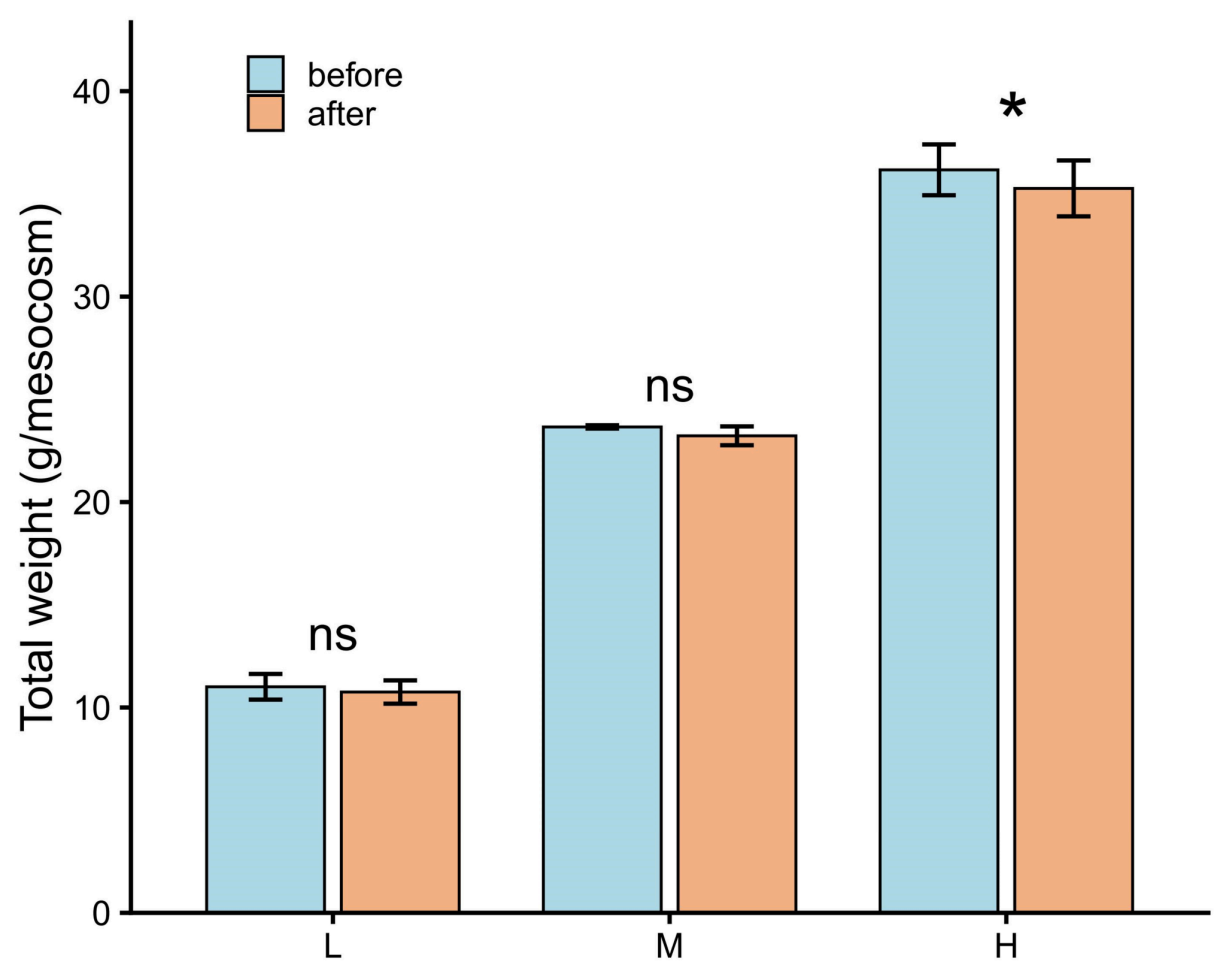
Changes in the total fresh biomass of golden apple snail between the beginning and end of the experiment. Values are mean ± SE (*n* = 3). L, M, and H represent the low-, medium-, and high-density treatments, respectively. “Before” and “after” indicate total snail fresh biomass on Day 0 and Day 20, respectively. Differences between sampling times within each treatment were evaluated using paired-sample *t*-tests. * indicates a significant difference between the two sampling times within the same density treatment (*p* < 0.05); ns, not significant.

Analysis of snail growth showed that total fresh biomass did not differ significantly between the beginning and end of the experiment in either the low-density treatment (*t* = −4.187, df = 2, *p* = 0.053) or the medium-density treatment (*t* = −0.852, df = 2, *p* = 0.484). In contrast, total snail fresh biomass in the high-density treatment was significantly lower at the end of the experiment than at the beginning (*t* = −5.909, df = 2, *p* = 0.027; Figure 1).

**Figure 2:**
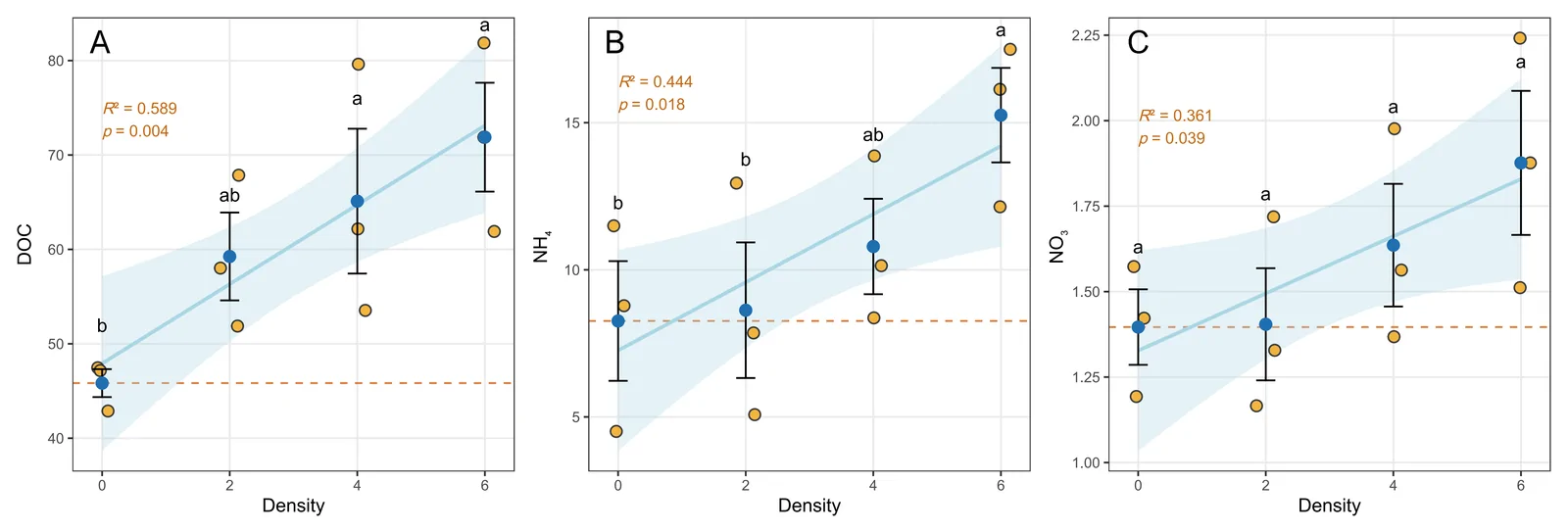
Effects of golden apple snail density on (**A**) dissolved organic carbon (DOC), (**B**) ammonium nitrogen (NH_4_^+^-N), and (**C**) nitrate nitrogen (NO_3_^−^-N) in flooded paddy soil. Yellow points represent individual mesocosms, blue points and error bars represent means ± SE (*n* = 3), solid lines show simple linear regressions with 95% confidence intervals, and orange dashed lines indicate control means. Treatment effects were evaluated using one-way ANOVA followed, when appropriate, by Fisher’s protected LSD test without multiplicity adjustment. Different lowercase letters indicate significant pairwise differences (*p* < 0.05).

Soil dissolved organic carbon (DOC), ammonium nitrogen (NH_4_^+^-N), and nitrate nitrogen (NO_3_^−^-N) generally increased with increasing snail density (Figure 2).

Linear regression analysis showed a significant positive relationship between snail density and soil DOC concentration (*R*^2^ = 0.589, *p* = 0.004). DOC concentrations in the medium- and high-density treatments were significantly higher than those in the snail-free control (CK vs. H: *p* = 0.009; CK vs. M: *p* = 0.035), whereas the low-density treatment did not differ significantly from the other treatments (*p* > 0.05).

Soil NH_4_^+^-N concentration also increased significantly with snail density (*R*^2^ = 0.444, *p* = 0.018). The high-density treatment had a significantly higher NH_4_^+^-N concentration than the control and low-density treatments (H vs. CK: *p* = 0.032; H vs. L: *p* = 0.040), whereas the medium-density treatment did not differ significantly from the other treatments (*p* > 0.05).

A significant positive relationship was also observed between snail density and soil NO_3_^−^-N concentration (*R*^2^ = 0.361, *p* = 0.039). Although NO_3_^−^-N generally increased with snail density, pairwise comparisons revealed no significant differences among the four density treatments (*p* > 0.05).

### 3.2. Effects of Golden Apple Snail on Soil Enzyme Activities

**Figure 3:**
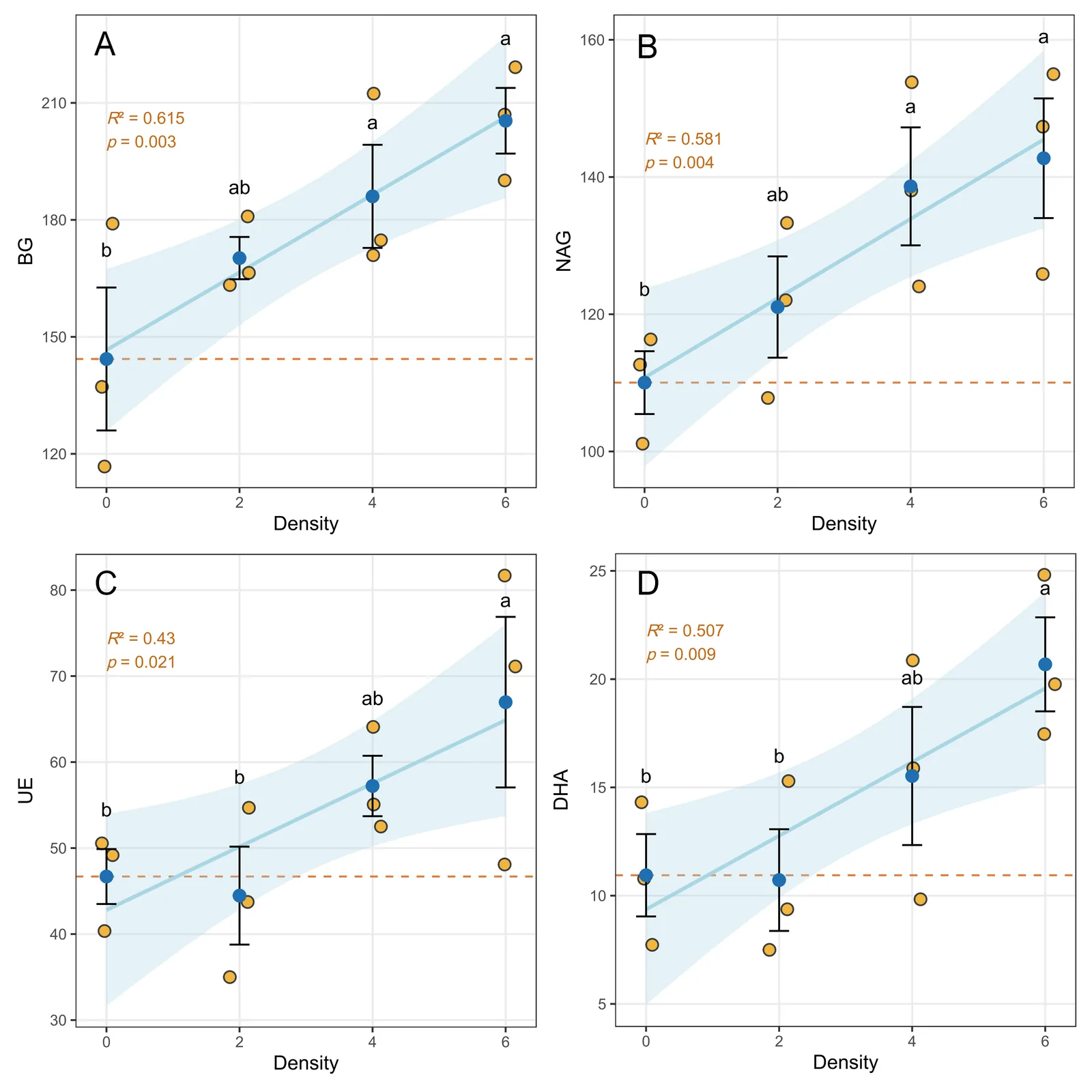
Effects of golden apple snail density on (**A**) β-glucosidase (BG), (**B**) N-acetyl-β-D-glucosaminidase (NAG), (**C**) dehydrogenase (DHA), and (**D**) urease (UE) activities in flooded paddy soil. Graphical elements are defined in Figure 2. Density relationships were evaluated using simple linear regression. Treatment effects were evaluated using one-way ANOVA followed, when the omnibus test was significant, by Fisher’s protected LSD test without multiplicity adjustment. Different lowercase letters indicate significant pairwise differences (*p* < 0.05).

The activities of β-glucosidase (BG), N-acetyl-β-D-glucosaminidase (NAG), urease (UE), and dehydrogenase (DHA) generally increased with increasing snail density (Figure 3).

BG activity was significantly and positively related to snail density (*R*^2^ = 0.615, *p* = 0.003). BG activities in the medium- and high-density treatments were significantly higher than those in the snail-free control (CK vs. H: *p* = 0.008; CK vs. M: *p* = 0.044), whereas the low-density treatment did not differ significantly from the other treatments *(p* > 0.05).

NAG activity also increased significantly with snail density (*R*^2^ = 0.581, *p* = 0.004). The medium- and high-density treatments had significantly higher NAG activities than the control (CK vs. H: *p* = 0.009; CK vs. M: *p* = 0.035), whereas the low-density treatment did not differ significantly from the other treatments (*p* > 0.05).

UE activity was significantly and positively associated with snail density (*R*^2^ = 0.430, *p* = 0.021). UE activity in the high-density treatment was significantly higher than that in the control and low-density treatments (CK vs. H: *p* = 0.049; L vs. H: *p* = 0.033). The medium-density treatment exhibited an intermediate value and did not differ significantly from the other treatments (*p* > 0.05).

DHA activity similarly increased with snail density, with a significant positive linear relationship between the two variables (*R*^2^ = 0.507, *p* = 0.009). DHA activity in the high-density treatment was significantly higher than that in the control and low-density treatments (CK vs. H: *p* = 0.023; L vs. H: *p* = 0.021), whereas the medium-density treatment did not differ significantly from the other treatments (*p* > 0.05).

### 3.3. Effects of Golden Apple Snail on Soil Bacterial Diversity and Community Composition

#### 3.3.1. Taxonomic Composition

Taxonomic annotation identified bacterial sequences belonging to 57 phyla, 147 classes, 315 orders, 476 families, and 883 genera across all samples.

**Figure 4:**
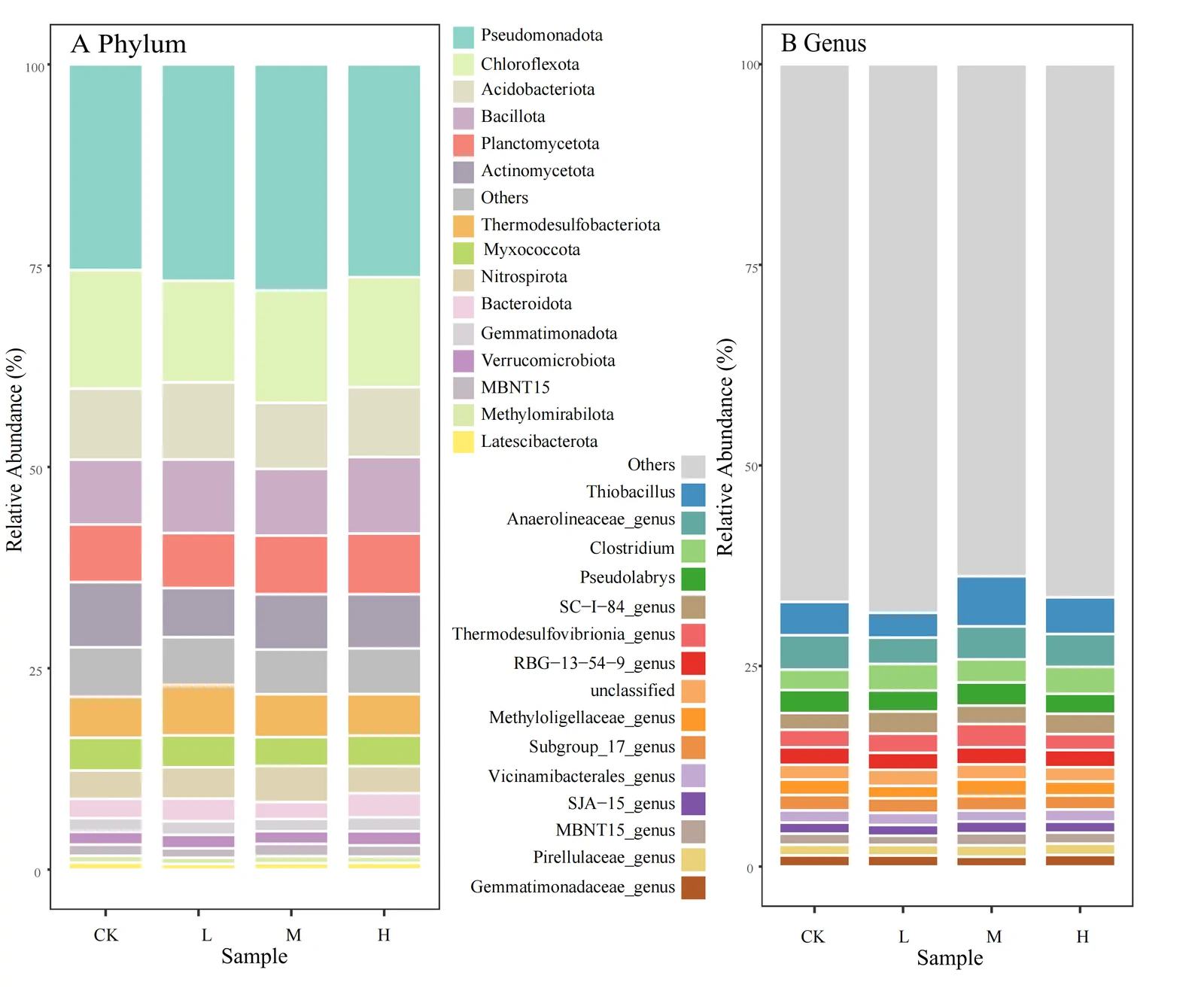
Bacterial community composition under different snail-density treatments at the (**A**) phylum and (**B**) genus levels. CK denotes the snail-free control; L, M, and H are defined in Figure 1. Colors represent bacterial taxa, bar heights indicate relative abundances, and “Others” comprises taxa not displayed individually. Differences in the relative abundances of individual taxa among treatments were evaluated using one-way ANOVA followed, where applicable, by Fisher’s LSD test (*p* < 0.05).

At the phylum level, bacterial community composition was generally similar among treatments. The dominant phyla were Pseudomonadota (26.73%), Chloroflexota (13.74%), Acidobacteriota (8.83%), Bacillota (8.74%), and Planctomycetota (7.21%). The relative abundances of the major bacterial phyla did not differ significantly among snail-density treatments (*p* > 0.05; Figure 4).

At the genus level, bacterial communities also exhibited similar compositional patterns among treatments. The dominant genus-level taxa included *Thiobacillus* (4.68%), an unclassified genus within Anaerolineaceae (4.03%), *Clostridium* (3.01%), and *Pseudolabrys* (2.70%). No significant differences were detected in the relative abundances of the dominant genera among treatments (*p* > 0.05).

#### 3.3.2. Bacterial α-Diversity

Across the 12 endpoint samples, 231,458–288,076 raw reads were generated per sample, of which 49,841–61,762 non-chimeric reads were retained after sequence processing. The OTU table was subsequently rarefied to 49,697 reads per sample for downstream analyses. Good’s coverage ranged from 0.9452 to 0.9504, indicating that approximately 94.5–95.0% of the bacterial diversity represented in the processed dataset was sampled. Sample-specific sequencing depths and Good’s coverage values are provided in Supplementary Table S1.

All three α-diversity indices showed an overall tendency to increase with increasing snail density. However, the linear relationships between snail density and the Chao1 richness index (*F*_1,10_ = 1.074, *p* = 0.325) and Shannon diversity index (*F*_1,10_ = 3.688, *p* = 0.084) were not significant. Neither index differed significantly among density treatments (*p* > 0.05; Table 1).

The Simpson index showed no significant linear trend with snail density. Post hoc comparisons detected a significant but small difference between the high-density treatment and the snail-free control (0.9977 vs. 0.9955; *p* = 0.041), while the other treatments did not differ significantly.

### 3.4. Environmental Factors Associated with Bacterial Community Variation

#### 3.4.1. Relationships Between Soil Nutrients and Enzyme Activities

**Figure 5:**
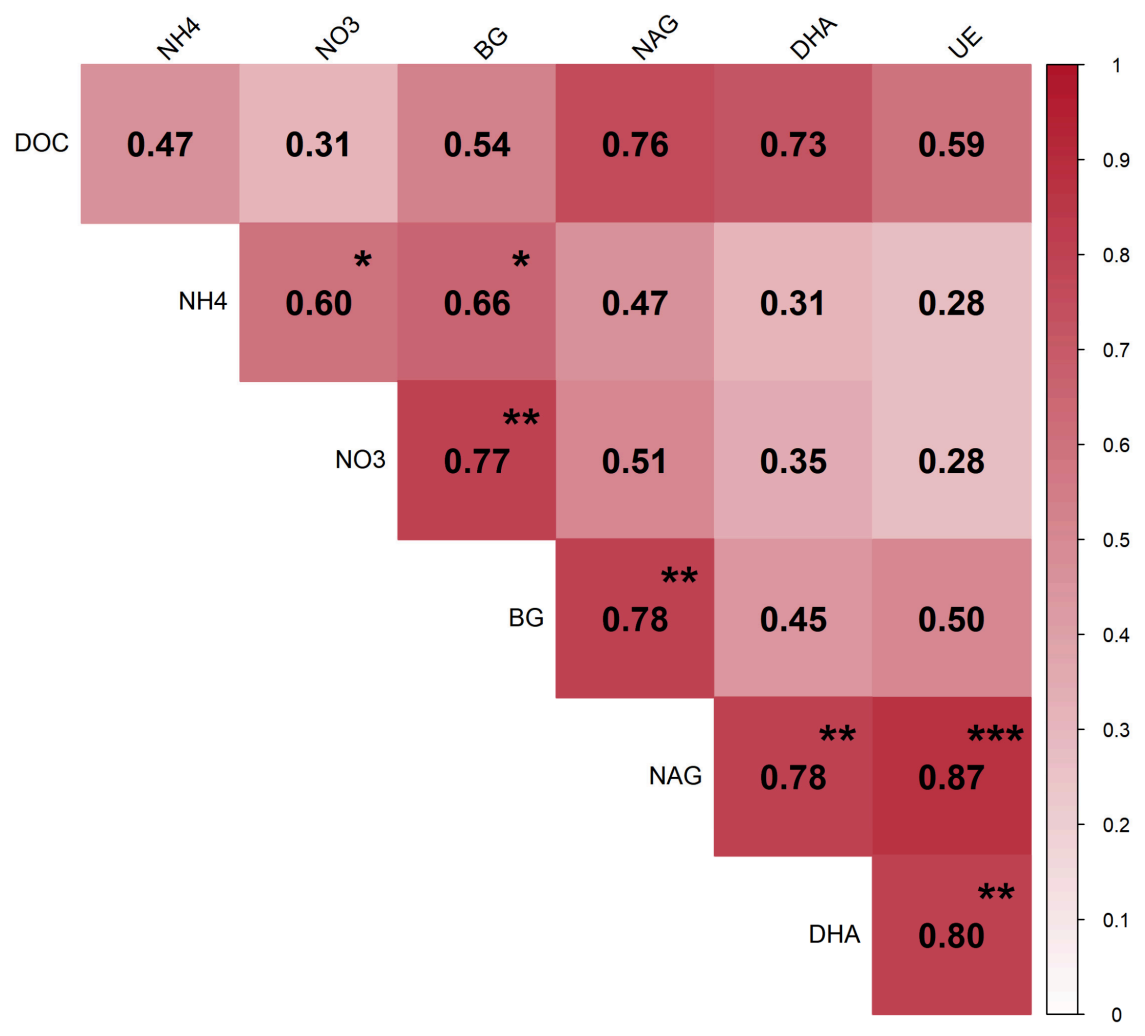
Spearman correlations among soil nutrient variables and enzyme activities. Cell values are Spearman’s rank correlation coefficients (*r_s_*), with darker red indicating stronger positive correlations. Variable abbreviations are defined in Figure 2 and Figure 3. Pairwise associations were evaluated using Spearman’s rank correlation test. *, **, and *** indicate *p* < 0.05, *p* < 0.01, and *p* < 0.001, respectively.

Spearman correlation analysis was performed to examine coordinated variation among DOC, NH_4_^+^-N, NO_3_^−^-N, BG, NAG, DHA, and UE. Overall, soil nutrient variables and enzyme activities were positively correlated with one another (Figure 5).

NH_4_^+^-N was significantly and positively correlated with NO_3_^−^-N (*r_s_* = 0.60, *p* = 0.039) and BG activity (*r_s_* = 0.66, *p* = 0.020). NO_3_^−^-N was strongly and positively correlated with BG activity (*r_s_* = 0.77, *p* = 0.003). BG activity was also positively correlated with NAG activity (*r_s_* = 0.78, *p* = 0.003).

NAG activity was positively correlated with both DHA activity (*r_s_* = 0.78, *p* = 0.003) and UE activity (*r_s_* = 0.87, *p* = 0.001). A strong positive correlation was also observed between DHA and UE activities (*r_s_* = 0.80, *p* = 0.002). Although the remaining pairwise relationships were positive, they were not statistically significant (*p* > 0.05).

#### 3.4.2. Relationships Between Bacterial Community Structure and Environmental Factors

**Figure 6:**
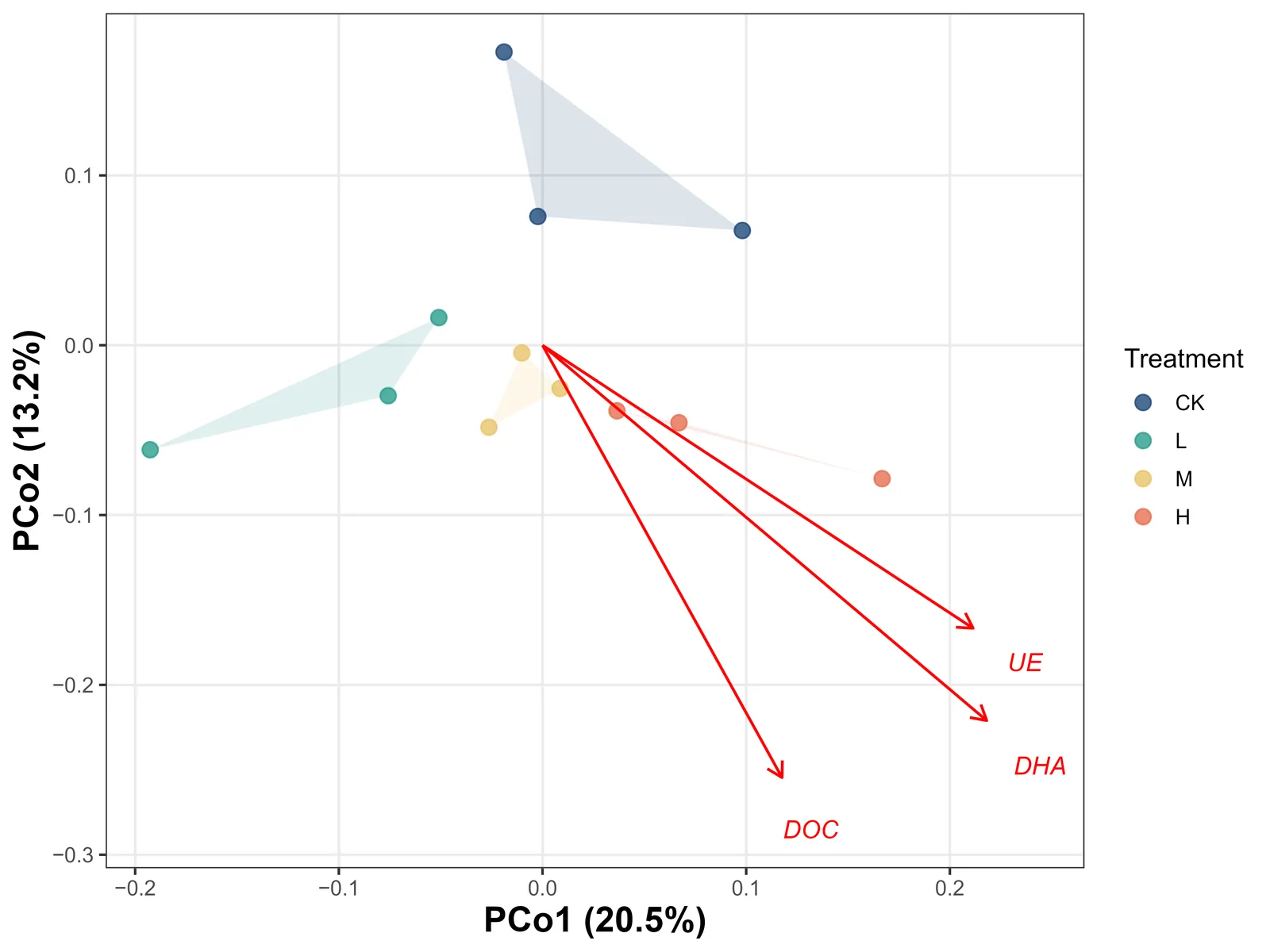
Principal coordinate analysis (PCoA) of bacterial communities based on Bray–Curtis dissimilarities. Points represent independent mesocosms, and colored polygons are convex hulls connecting the three replicates within each treatment. Treatment differences were tested using PERMANOVA with 999 permutations, and homogeneity of multivariate dispersions was assessed using PERMDISP with 9999 permutations. Environmental associations were evaluated using envfit with 999 permutations; red vectors indicate significant associations (*p* < 0.05) for dissolved organic carbon (DOC), dehydrogenase activity (DHA), and urease activity (UE). PCo1 and PCo2 explain 20.5% and 13.2% of the variation, respectively.

Principal coordinates analysis based on Bray–Curtis dissimilarities showed that PCo1 and PCo2 explained 20.5% and 13.2% of the variation in bacterial community structure, respectively, accounting for 33.7% of the total variation (Figure 6).

PERMANOVA indicated that bacterial community structure differed significantly among snail-density treatments (*F*_3,8_ = 1.505, *p* = 0.001). Multivariate dispersion did not differ significantly among treatments (*F*_3,8_ = 3.430, *p* = 0.0681), indicating that the PERMANOVA result was unlikely to be explained solely by differences in within-treatment dispersion. Samples from different treatments exhibited a gradual distribution pattern in the ordination space. Control samples were mainly distributed along the positive direction of PCo2, low-density samples tended to occur along the negative direction of PCo1, and medium- and high-density samples generally shifted toward the positive direction of PCo1 and the negative direction of PCo2. This pattern indicated a directional shift in bacterial community structure along the snail-density gradient.

Environmental fitting showed that DOC (*r*^2^ = 0.534, *p* = 0.028), UE activity (*r*^2^ = 0.654, *p* = 0.005), and DHA activity (*r*^2^ = 0.492, *p* = 0.046) were significantly associated with bacterial community ordination. Their vectors generally pointed toward the regions occupied by samples from the medium- and high-density treatments.

The DOC vector was primarily oriented toward the negative direction of PCo2, whereas the UE and DHA vectors extended toward both the positive direction of PCo1 and the negative direction of PCo2. In contrast, NH_4_^+^-N, NO_3_^−^-N, BG, and NAG were not significantly associated with bacterial community structure (*p* > 0.05).

## 4. Discussion

### 4.1. Golden Apple Snail Activity Alters Labile Carbon and Nitrogen Pools in Flooded Paddy Soil

In the present study, soil DOC, NH_4_^+^-N, and NO_3_^−^-N concentrations all increased with increasing snail density. This finding indicates that, even in the absence of rice-derived rhizosphere inputs and external food sources, living golden apple snails can directly alter the nutrient conditions of flooded paddy soil. Previous studies have demonstrated that golden apple snail releases ammonium into the surrounding water and that its activity can increase carbon, nitrogen, and phosphorus concentrations in wetland water bodies [6,19]. Research on other benthic gastropods has further shown that snail bioturbation can modify oxygen and nutrient fluxes across the sediment–water interface [20]. Therefore, the accumulation of labile carbon and nitrogen observed in this study was likely associated with the combined effects of metabolic excretion, egestion, and snail-induced disturbance of the surface soil.

The density-dependent increase in NH_4_^+^-N was broadly consistent with the findings of Chen et al. [14] in coastal saline agricultural soil, suggesting that ammonium accumulation may represent an important pathway through which living golden apple snail modifies nutrient availability in agricultural soils. Previous carcass-amendment studies have also reported increases in soil carbon and nitrogen availability [11,12,13]. However, these studies involved discrete organic-matter additions, whereas the present experiment examined the short-term effects of continuous activity by living snails. This difference in input form should be considered when comparing nutrient responses between the two experimental systems.

Soil DOC concentration increased with snail density in the present study, whereas Chen et al. [14] observed a reduction in DOC following the introduction of golden apple snail into saline soil. This contrast suggests that the effects of the snail on soil carbon dynamics are strongly dependent on environmental context. In the study by Chen et al. [14], salinity substantially influenced the bacterial community, microbial biomass, and soil enzyme activities. Oxygen availability is also a major regulator of the pathways and rates of organic carbon mineralization in flooded soils [21]. The present experiment used nonsaline paddy soil and lasted only 20 days. Under these conditions, the addition of soluble organic compounds through snail metabolism and egestion, together with the release of soil-derived organic matter through surface bioturbation, may have exceeded microbial consumption during the incubation period, resulting in net DOC accumulation.

Soil NO_3_^−^-N also increased with snail density. Bioturbation by benthic gastropods can alter oxygen exchange at the sediment–water interface [20], whereas enhanced oxygen availability in paddy soil can influence ammonia-oxidizing microorganisms, nitrogen-transforming enzyme activities, and the abundance of associated functional genes [22]. Snail movement and burrowing may therefore have modified localized redox and oxygen conditions at the soil-water interface, allowing NH_4_^+^-N release and partial nitrification to occur concurrently. In addition, both the quantity and composition of DOC can affect nitrification by selectively promoting particular bacterial groups [23]. Nevertheless, because soil redox potential, dissolved oxygen, nitrification rates, and ammonia-oxidation functional genes were not measured, the specific nitrogen transformation pathways responsible for the observed increase in NO_3_^−^-N cannot be determined from the present data.

Total snail biomass in the high-density treatment decreased significantly by the end of the experiment, which may indicate food limitation, intraspecific competition, or density-related stress in the absence of external food inputs. Therefore, nutrient accumulation in the high-density treatment cannot be attributed to an increase in snail biomass. Instead, it more likely resulted from the greater number of individuals per unit area and the associated enhancement of metabolic release, egestion, and soil disturbance. Taken together, these results represent short-term belowground responses under simplified conditions; the absence of rice plants and plant nutrient uptake may have contributed to nutrient accumulation, and the findings should not be interpreted as an agronomic benefit.

### 4.2. Accumulation of Labile Carbon and Nitrogen Enhances Soil Enzyme Activities Associated with Carbon and Nitrogen Cycling

The activities of BG, NAG, UE, and DHA increased significantly with increasing snail density, indicating that the effects of golden apple snail extended beyond changes in soil nutrient concentrations to biochemical processes involved in carbon and nitrogen transformation. BG and NAG participate in the hydrolysis of carbon- and nitrogen-containing organic compounds, respectively. UE catalyzes the hydrolysis of urea and the release of NH_4_^+^-N, whereas DHA is commonly used as an indicator of the overall oxidative and metabolic activity of viable soil microorganisms. Extracellular enzymes are central to microbial acquisition of carbon and nitrogen resources, and their activities are jointly regulated by substrate availability, microbial biomass, and microbial community composition [24,25,26].

The positive enzyme responses observed in this study are consistent with the general dependence of extracellular enzyme activities on substrate and nutrient availability [24,25,26,27]. Studies involving organic inputs have shown that changes in DOC and nutrient availability are often accompanied by shifts in soil enzyme activities and microbial processes [27]. Because no plant residues, organic fertilizer, or supplemental feed were added in the present experiment, the observed increases in enzyme activities indicate that changes in soil biochemical activity occurred under inputs associated with living-snail activity.

Spearman correlation analysis further revealed several significant positive relationships among inorganic nitrogen concentrations and the activities of BG, NAG, UE, and DHA, indicating coordinated changes in nutrient availability and microbial enzymatic activity. Labile carbon and nitrogen released through snail activity may have stimulated microbial growth and the production of resource-acquisition enzymes. In turn, enhanced enzymatic decomposition may have promoted the hydrolysis of soil organic matter and microbial residues, potentially reinforcing the supply of available substrates. Previous studies have similarly demonstrated close linkages among soil carbon and nitrogen availability, microbial functional gene composition, and extracellular enzyme activities [25,26].

The increase in UE activity may also be associated with the accumulation of NH_4_^+^-N. Studies in paddy soils have shown that UE and other enzymes involved in nitrogen transformation are closely related to microbial biomass and the abundance of nitrogen-cycling functional genes [22]. Nevertheless, the enzyme activities measured in the present study represent potential activities determined under standardized assay conditions rather than in situ process rates. Consequently, the observed increases cannot be interpreted as direct evidence that actual carbon mineralization or net nitrogen mineralization rates increased to the same extent under microcosm conditions.

Despite this limitation, the coordinated responses of nutrient pools and enzyme activities show that snail-induced changes in resource availability substantially altered the microbial metabolic environment. These biochemical changes may, in turn, have contributed to the subsequent adjustment of the soil bacterial community.

### 4.3. Bacterial Community Structure Shifts Despite the Stability of Dominant Taxa

Pseudomonadota, Chloroflexota, Acidobacteriota, Bacillota, and Planctomycetota were the dominant bacterial phyla detected in the flooded paddy soil. This taxonomic profile is broadly consistent with bacterial community compositions previously reported for paddy soils [28,29,30,31,32,33]. The relative abundances of the major phyla and genera did not differ significantly among snail-density treatments, indicating that 20 days of snail activity did not cause large-scale replacement of the dominant bacterial taxa.

Long-term fertilization studies have shown that pronounced community reorganization at the phylum level generally occurs in response to cumulative changes in soil nutrient availability, pH, organic matter content, and other environmental properties over multiple years [28,29,31,32,33]. In comparison, the disturbance period in the present study was relatively short, and the magnitude of material input from living snails was limited. These changes may therefore have been insufficient to override the environmental filtering processes that maintain the dominant bacterial assemblages in flooded paddy soil.

Neither Chao1 richness nor Shannon diversity differed significantly among treatments, indicating no major change in bacterial α-diversity during the 20-day incubation. Although the Simpson index was significantly higher in the high-density treatment than in the snail-free control, the absolute difference was small (0.9977 vs. 0.9955). This small difference suggests only a subtle shift in community evenness or dominance rather than a substantial change in overall bacterial α-diversity.

This subtle response differs from the decline in bacterial richness reported following high-dose additions of golden apple snail carcasses [12], possibly reflecting differences in the form and intensity of snail-derived inputs. However, because the quantity and temporal pattern of these inputs were not directly measured in the present study, the underlying mechanism cannot be determined conclusively.

Although the dominant taxa and most α-diversity indices remained relatively stable, the PCoA results revealed a directional distribution of samples along the snail-density gradient. This pattern suggests that snail activity caused relatively small but coordinated changes across multiple bacterial taxa, collectively resulting in a detectable shift in overall community structure. Multivariate ordination integrates relative abundance information across all detected taxa and is therefore often more sensitive to changes in low-abundance groups and subtle shifts within dominant lineages than comparisons conducted at a single taxonomic level [34].

At the genus level, *Thiobacillus*, an unclassified genus within Anaerolineaceae, *Clostridium*, and *Pseudolabrys* were relatively abundant. Because functional genes and metagenomes were not analyzed, their specific ecological roles cannot be inferred solely from taxonomic identity. Nevertheless, previous research on flooded paddy soils has shown that oxygen availability and the stage of organic matter decomposition can jointly select for bacterial groups associated with fermentation, organic carbon transformation, and nitrogen and sulfur cycling [21,35]. The relative stability of these dominant genera across treatments suggests that, although snail activity changed resource availability and microbial metabolic intensity, it did not fundamentally alter the prevailing environmental selection regime of the flooded soil.

Overall, the bacterial community exhibited a response characterized by the relative stability of dominant taxa accompanied by adjustments in community evenness and overall structure. This pattern suggests that the effects of golden apple snail on soil bacterial communities were more likely mediated through changes in soil resource conditions and microbial metabolic processes than through the direct replacement of dominant bacterial groups.

### 4.4. DOC and Microbial Metabolic Processes Are Important Correlates of Bacterial Community Variation

Environmental fitting showed that DOC, UE, and DHA were significantly associated with bacterial community ordination, and their vectors generally pointed toward samples from the medium- and high-density treatments. These relationships were consistent with the density-dependent responses of soil nutrients and enzyme activities, suggesting that labile carbon availability, nitrogen hydrolysis potential, and overall microbial metabolic activity may represent important links between snail activity and bacterial community variation.

DOC is highly mobile and often readily bioavailable, allowing it to serve as an immediate energy and carbon source for heterotrophic microorganisms. By altering resource availability and competition among microbial groups, changes in DOC quantity and composition can substantially influence bacterial community assembly. Previous research has shown that dissolved organic matter can selectively favor particular bacterial taxa and affect nitrogen transformation processes [33]. Changes in DOC resulting from different organic inputs are also frequently accompanied by adjustments in bacterial community structure and extracellular enzyme activities [27]. In the present study, DOC increased with snail density and was significantly associated with bacterial community ordination, indicating that the increased supply of labile carbon caused by snail activity may have provided an important environmental basis for the observed directional shift in community structure.

UE and DHA reflect different but complementary aspects of soil microbial functioning. UE represents the potential for urea hydrolysis and ammonium release, whereas DHA reflects the overall respiratory and redox activity of viable microbial cells. Their significant relationships with bacterial community structure, together with their close correlations with NAG and other enzymes, suggest that community variation was not associated with a single nutrient pool alone. Rather, it was linked to the coordinated enhancement of organic matter decomposition, nitrogen release, and microbial metabolic activity.

Studies integrating functional genes and enzyme activities have demonstrated close relationships between microbial functional composition and soil carbon-degrading enzymes [26]. In paddy soils, nitrogen-transforming enzyme activities, microbial biomass, and relevant functional genes can also respond jointly to environmental change [22]. Long-term organic matter inputs can likewise reshape paddy soil bacterial communities by modifying resource availability and physicochemical conditions [28,29,31]. The present study extends these observations by showing that changes in DOC and microbial metabolic activity associated with living golden apple snail were closely associated with bacterial community structure.

Although NH_4_^+^-N and NO_3_^−^-N increased with snail density, neither variable was significantly associated with bacterial community ordination. Concentrations of inorganic nitrogen at a single sampling time represent the net outcome of several simultaneous processes, including mineralization, nitrification, denitrification, microbial immobilization, diffusion, and exchange with the overlying water. Endpoint concentrations may therefore not directly reflect the temporal dynamics of the microbial groups responsible for these transformations. Moreover, the present study included only 12 endpoint samples, which limited the statistical power of environmental fitting for detecting relatively weak relationships. Covariation among DOC, inorganic nitrogen, and enzyme activities may also have obscured the independent contribution of individual variables.

Accordingly, DOC, UE, and DHA should be interpreted as environmental indicators closely associated with bacterial community variation rather than as independently verified causal drivers. Experimental manipulation of individual variables, together with measurements of microbial biomass, process rates, functional genes, and temporal community dynamics, would be required to establish causal pathways.

Collectively, the results are consistent with the following sequence of responses: increasing snail density enhanced metabolic inputs, egestion, and surface-soil bioturbation, which were accompanied by the accumulation of DOC and inorganic nitrogen; changes in labile carbon and nitrogen availability coincided with increased activities of BG, NAG, UE, and DHA; and the resulting changes in resource availability and microbial metabolic conditions were subsequently associated with adjustments in bacterial diversity and overall community structure. Thus, the ecological effects of golden apple snail are not restricted to rice consumption and nutrient release into the water column but can also extend to the soil-microbe interface of flooded paddy ecosystems.

These associations were observed during a 20-day microcosm incubation without rice plants, a design that isolated belowground snail effects but excluded plant nutrient uptake and direct herbivory. Future work will extend the experiment to field conditions with rice to determine whether these soil–microbial responses persist and how they interact with seedling damage and crop performance.

## 5. Conclusions

This study demonstrated that, under controlled flooded paddy soil conditions without rice plants or external food inputs, increasing golden apple snail density was accompanied by higher soil DOC, NH_4_^+^-N, and NO_3_^−^-N concentrations and increased activities of BG, NAG, UE, and DHA. The dominant bacterial taxa remained largely stable, whereas overall bacterial community structure differed among density treatments. DOC, UE, and DHA were significantly associated with bacterial community variation. These results indicate that living golden apple snail density is linked to short-term changes in soil nutrient status, biochemical activity, and bacterial community organization, but do not by themselves establish the mechanisms connecting these responses.

Overall, the observed responses extend the recognized ecological effects of golden apple snail invasion beyond rice herbivory and water-column nutrient changes to belowground soil processes. Because carbon and nitrogen transformation rates, microbial functional genes, oxygen and redox dynamics, and temporal response trajectories were not directly measured, the relationships among nutrient availability, enzyme activities, and bacterial community variation should be interpreted as associations rather than a demonstrated sequential mechanism. Long-term field validation is still needed, and these belowground responses do not support introducing this serious rice pest into fields.

## Figures and Tables

**Table 1:** Bacterial α-diversity indices in flooded paddy soil under different snail density treatments. Values are presented as the mean ± SE (n = 3).

	CK	L	M	H
Chao1	9861.10 ± 148.98 ^a^	10,049.95 ± 127.92 ^a^	9907.53 ± 108.93 ^a^	10,080.13 ± 28.01 ^a^
Shannon	7.68 ± 0.09 ^a^	7.81 ± 0.03 ^a^	7.81 ± 0.04 ^a^	7.83 ± 0.04 ^a^
Simpson	0.9955 ± 0.0012 ^b^	0.9975 ± 0.0003 ^ab^	0.9973 ± 0.002 ^ab^	0.9977 ± 0.0003 ^a^

Note: CK, L, M, and H represent the snail-free control and the low-, medium-, and high-density treatments, respectively. Different lowercase letters within the same row indicate significant differences among treatments (*p* < 0.05).

## Data Availability

The raw full-length 16S rRNA gene amplicon sequencing data generated in this study are openly available in the NCBI Sequence Read Archive under BioProject accession number PRJNA1502501. The remaining data supporting the findings of this study are available from the corresponding author upon reasonable request.

## Supplementary Materials

The following supporting information can be downloaded at: https://www.mdpi.com/article/10.3390/microorganisms14091895/s1, Table S1: Sequencing depth and Good’s coverage for individual soil samples.

## Abbreviations

The following abbreviations are used in this manuscript:

DOC	Dissolved organic carbon
NH_4_^+^-N	Ammonium nitrogen
NO_3_^−^-N	Nitrate nitrogen
TN	Total nitrogen
NAG	N-acetyl-β-D-glucosaminidase
UE	Urease
DHA	Dehydrogenase
BG	β-glucosidase
OTU	Operational taxonomic unit
ANOVA	One-way analysis of variance
LSD	Fisher’s least significant difference
PCoA	Principal coordinates analysis
PERMANOVA	Permutational multivariate analysis of variance
