# Supplementary material for: Density-Dependent Effects of Invasive Pomacea canaliculata on Nutrient Status, Enzyme Activities, and Bacterial Community Structure in Flooded Paddy Soil Microcosms
Source: Microorganisms. 2026 Aug 26;14(9):1895. doi: 10.3390/microorganisms14091895 (PMC13609590; doi:10.3390/microorganisms14091895)
Supplement: Supplementary file 1 [file microorganisms-14-01895-s001.zip › microorganisms-4506374-supplementary.pdf]

Table S1 Sequencing depth and Good’ s coverage for individual soil samples

Sample ID	Treatment	Non-chimeric reads	Reads after rarefaction	Good’s coverage
CK1	snail-free control	51,289	49,697	0.9485281
CK2	snail-free control	51,707	49,697	0.9473208
CK3	snail-free control	49,841	49,697	0.9476427
L1	low-density treatments	55,805	49,697	0.9503793
L2	low-density treatments	50,260	49,697	0.9493330
L3	low-density treatments	59,124	49,697	0.9469988
M1	medium-density treatments	61,762	49,697	0.9493732
M2	medium-density treatments	57,293	49,697	0.9476025
M3	medium-density treatments	52,694	49,697	0.9473409
H1	high-density treatments	59,605	49,697	0.9470994
H2	high-density treatments	56,226	49,697	0.9476830
H3	high-density treatments	52,913	49,697	0.9451677

Note: Raw reads represent the sequences obtained before quality filtering. Non-chimeric reads represent the sequences retained after quality filtering, primer removal, and chimera removal. The OTU table was rarefied to 49,697 reads per sample before diversity and community analyses. CK, L, M, and H denote the snail-free control and the low-, medium-, and high-density treatments, respectively.
